# Inhibitory Peptide of Soluble Guanylyl Cyclase/Trx1 Interface Blunts the Dual Redox Signaling Functions of the Complex

**DOI:** 10.3390/antiox12040906

**Published:** 2023-04-10

**Authors:** Chuanlong Cui, Ping Shu, Tanaz Sadeghian, Waqas Younis, Hong Li, Annie Beuve

**Affiliations:** 1 School of Graduate Studies, Newark Health Science Campus, Rutgers, The State University of New Jersey, Newark, NJ 07103, USA; 2 Department of Physiology, Pharmacology and Neurosciences, New Jersey Medical School, Rutgers, The State University of New Jersey, Newark, NJ 07103, USA; 3 Center for Advanced Proteomics Research, Department of Microbiology, Biochemistry and Molecular Genetics, New Jersey Medical School, Rutgers, The State University of New Jersey, Newark, NJ 07103, USA

**Keywords:** thioredoxin, oxidative stress, NO, transnitrosation, mimetic peptide, soluble guanylyl cyclase, protein–protein interaction, S-nitrosylation, reductase

## Abstract

Soluble guanylyl cyclase (GC1) and oxido-reductase thioredoxin (Trx1) form a complex that mediates two NO signaling pathways as a function of the redox state of cells. Under physiological conditions, reduced Trx1 (rTrx1) supports the canonical NO-GC1-cGMP pathway by protecting GC1 activity from thiol oxidation. Under oxidative stress, the NO-cGMP pathway is disrupted by the S-nitrosation of GC1 (addition of a NO group to a cysteine). In turn, SNO-GC1 initiates transnitrosation cascades, using oxidized thioredoxin (oTrx1) as a nitrosothiol relay. We designed an inhibitory peptide that blocked the interaction between GC1 and Trx1. This inhibition resulted in the loss of a) the rTrx1 enhancing effect of GC1 cGMP-forming activity in vitro and in cells and its ability to reduce the multimeric oxidized GC1 and b) GC1’s ability to fully reduce oTrx1, thus identifying GC1 novel reductase activity. Moreover, an inhibitory peptide blocked the transfer of S-nitrosothiols from SNO-GC1 to oTrx1. In Jurkat T cells, oTrx1 transnitrosates procaspase-3, thereby inhibiting caspase-3 activity. Using the inhibitory peptide, we demonstrated that S-nitrosation of caspase-3 is the result of a transnitrosation cascade initiated by SNO-GC1 and mediated by oTrx1. Consequently, the peptide significantly increased caspase-3 activity in Jurkat cells, providing a promising therapy for some cancers.

## 1. Introduction

Thioredoxin 1 (Trx1) is an oxido-reductase that was first described for its ability to reduce disulfide bonds and protect against oxidative stress through a mechanism that involves its reduced C^32^xxC^35^ cysteine (Cys) active center. As such, it is one of the most critical regulators of cellular redox and a key player in the antioxidant defense system in cells [1]. We previously showed that soluble guanylyl cyclase (GC1) could form a complex with Trx1, which, in turn, enhanced the NO-stimulated catalytic activity of GC1 in cells and in vitro [2]. The hundred-fold stimulation of cGMP production by NO requires the α and β subunits of GC1 to form a heterodimer. A Trx1-trap system and mutational analysis suggest that a mixed disulfide between C^32^ of the Trx1 active site and Cys 610 (C^610^) of the α subunit of GC1 is necessary for a Trx1-dependent increase of GC1 in response to NO stimulation [2,3]. We previously showed that the NO-stimulated activity of GC1 was reduced by the S-nitrosation of several Cys [4,5,6]. S-nitrosation (addition of a NO moiety to a Cys) is a posttranslational modification (PTM) that changes the properties of the proteins, including activity, localization, and interaction [7]. Interestingly, Trx1 can catalyze denitrosation, which is the removal of the nitroso group (SNO) from the Cys of a protein through a mechanism that involves its reduced C^32^xxC^35^ active center, as for disulfide reduction. As such, the mechanism of Trx1-dependent increases in GC1 activity could involve a reduction in oxidized thiols and/or the protection of GC1 from desensitization to NO by decreasing S-nitrosation of GC1 [2].

Trx1 is involved in the regulation of cellular S-nitrosation, not only via de-nitrosation but also via transnitrosation reactions. Transnitrosation by protein–protein interactions is the direct transfer of the nitroso group from the thiol on one protein to a target thiol on another protein, thus supporting a high degree of specificity for this PTM and its relevance as another form of NO signaling [8]. Trx1 transnitrosation requires Trx1 to be oxidized (oTrx1) with the formation of a disulfide bond between C^32^–C^35^ of its active site, while Cys73 (C^73^) is the main SNO donor, i.e., transferring its SNO to the thiol of the targeted protein [9]. We recently reported that the α subunit of GC1, in the absence of an active cGMP-forming α/β heterodimer, can itself transnitrosate more than 200 proteins under oxidative/nitrosative stress [10]. Remarkably, this high number of GC1’ SNO-targets is partially due to GC1’s ability to interact with and use oTrx1 as a mediator of the S-nitrosothiols transfer. We demonstrated that the SNO-GC1→oTrx1 transnitrosation reaction was unilateral, did not take place if Trx1 was in its reduced form (rTrx1), and that the SNO transfer involved C^610^ of the GC1-α subunit to C^73^ of oTrx1 [10]. As an example of the biological relevance of the transnitrosation reaction, we specifically identified a transnitrosation cascade initiated by SNO-GC1 that required oTrx1 and led to S-nitrosation of RhoA and the inhibition of its activity in cells.

Together, these findings suggest that the GC1/Trx1 complex differentially supports NO signaling, that is, cGMP production and nitrosation, as a function of the redox of cells. In addition, we showed that αC^610^ was involved in both the reduced and oxidized GC1/Trx1 complex formation and function. Our previous studies used mutants of Trx1 or an inhibitor of Trx1-reductase, thus altering the entire redox state of the cells and limiting the interpretation of the role of the GC1/Trx1 complex in cells. Likewise, these previous studies involved the deletion of the α subunit of GC1 or replacement of Cys 610 with a Ser (α^C610S^) with the caveat that such a mutation could affect the structure and activity of GC1. In order to (a) understand how a different GC1 and Trx1 interaction takes place under reduced and oxidative conditions, (b) characterize the complex properties, and (c) seek their biological relevance, we designed a set of mimetic peptides of the αC610 region to disrupt the two complexes without affecting the integrity of Trx1 and GC1 under both reduced and oxidized conditions.

## 2. Materials and Methods

### 2.1. Reagents

The purified recombinant human GC1 protein was purchased from Enzo life sciences (ALX-201-177-C010), and the Thioredoxin 1 (Trx1) protein was from Fitzgerald industries (30R-2985). FBS was from VWR (97068-085), the GC1 α antibody was from ThermoFisher (MA517086, Waltham, MA, USA), the GC1 β antibody was from Cayman chemical company (160897), Trx1 antibodies were from cell signaling (2298S and human-specific: 2285S). The caspase 3 antibody was purchased from Novus biologicals (NB100-56708), and the biotin antibody from Abcam (ab1227). The following reagents were purchased from Sigma (St. Louis, MI, USA): DTT (D0632), diamide (D3648), Amicon Ultra-0.5 centrifugal filter (UFC500324), Bradford reagent (B6916), PMSF (P7626), protease inhibitor cocktail (P8340), Etoposide (E1383), and the Caspase 3 activity assay kit (CASP3C-1KT). [α-^32^P]-GTP was from PerkinElmer (NEG006H250UC), S-nitrosoglutathione from Calbiochem (487920). The jetPRIME transfection kit was from Polyplus transfection (101000015), Diethylamine NONOate (DEA-NO) from Enzo life sciences (ALX-430-034-5005) and biotin-HPDP from ThermoFisher (21341). The Jurkat T cell line, clone E6-1 was purchased from ATCC (TIB-152).

### 2.2. Peptides Synthesis

Two sets of peptides were synthesized. The first set of three 11-amino acid peptides, synthesized by Epoch Life science, mimicked the sequence of amino acids in the proximity of, or overlapping, Cysteine 610 of the α subunit of GC1. A fourth scramble peptide was synthesized as a control. The sequences are provided in Figure S1 of the Supplementary Information. As the 3 peptides were efficient at disrupting the GC1/Trx1 complex in vitro, peptide 3 (inhibitory peptide) and the scramble peptide were modified (Abclonal Science, Wuhan, China) for cell penetration through the addition of a tat/penetrating hybrid peptide and FITC (Figure S2) [11].

### 2.3. Cell Culture and Transfection

COS-7 cells (ATCC) were cultured in a 6-well plate with a complete medium DMEM from Corning (10-013-CV, Somerville, MA, USA) supplemented with 10% FBS (heat inactivated) and Pen-Strep (ThermoFisher, 15140122) at 37 °C with 5% CO_2_. The cells were transfected with the wild-type GC1 α and β subunits from rats subcloned in a pCMV5 vector [2], using the jetPRIME kit. After 4 h, the medium was replaced with a complete medium supplemented with 10%FBS + P/S. After 24 h of transfection, the cells were washed twice and cultured in a serum-free medium for peptide treatment.

Jurkat cells were cultured in a 6-well plate at the density of 0.25 × 10^6^/mL in complete medium RPMI 1640 from Corning (10-040-CV) supplemented with 10% FBS and Pen-Strep and incubated at 37 °C with 5% CO_2_ for 24 h. The cells were collected by centrifuging at 1000× *g* for 5 min, washed twice with serum-free medium, and re-suspended in a 2 mL serum-free medium prior to peptide treatment.

### 2.4. Peptides Treatment and Assay of GC1 Activity in a Purified System

Each peptide from set 1 (Figure S1) was dissolved in 70% DMSO to obtain a 10 mM stock solution and was further diluted with H_2_O to have a 250 μM working stock. In each reaction tube, 0.25 μg of human recombinant GC1 was mixed with 0.15 μg of Trx1 (molar ratio GC1:Trx1 ~ 1:7) or not (to control the effect of peptides on GC1 activity). In each tube of the GC1 and Trx1 mixture or GC1 alone, 5 μL of each peptide was added in a 50 μL final volume of 50 mM HEPES, pH 8.0. The final concentration of each peptide was 25 μM. The molar ratio of GC1 to each peptide was 1:800. The tubes were incubated on ice for 20 min in the dark. After incubation, the samples were subjected to a GC1 activity assay under basal and NO-stimulated conditions. The GC1 activity was measured by the conversion of [α-^32^P]-cGMP from [α-^32^P]-GTP, as previously described [12]. A fifty μL reaction mix containing 50 mM HEPES, pH 8.0, 5 mM MgCl_2_, and 0.5 mM GTP was added to each sample and incubated at 30 °C for 5 min. DEA-NO (1 μM final concentration) was used as the NO donor to stimulate GC1 activity. The reaction was stopped by adding 500 μL of 120 mM zinc acetate, followed by the addition of 500 μL of 144 mM sodium carbonate. [α-^32^P]-cGMP was separated using alumina columns. The recovery rate was calculated using 100 μL of cGMP-^3^H. This specific activity was first expressed in nmol cGMP·min^−1^·mg^−1^ (Figure S3); then, these values were expressed as a fold change normalized to GC1 NO-stimulated activity alone. *n* = 4 independent experiments were performed in duplicate.

### 2.5. Peptides Treatment and Assay of GC1 Activity in COS-7 Cells

After 24 h of transfection with WT GC1, COS-7 cells were either treated with 70% DMSO (0.35% final), the selected inhibitory peptide or scramble peptide at 37 °C for 1 h in a serum-free medium. The final concentration of each peptide was 5–10 μM (sequences are provided in Figure S2a). After 1 h incubation, the cells were washed twice and incubated in the complete medium at 37 °C for 24 h. After 24 h incubation, the cells were washed 3 times with ice-cold PBS and sonicated in homogenization buffer containing 50 mM HEPES, pH 8.0 and 150 mM NaCl supplemented with PMSF and a protease inhibitor cocktail. The protein concentration of each sample was measured using the Bradford method. A total of 20 μg of protein in each sample was used for GC1 activity assay under both basal and NO-stimulated conditions. The GC1 activity assay was conducted as above. Prior to lysis, coverslips were collected at 12 h and 24 h to visualize FITC fluorescence and assess peptide penetration (Figure S2b). Conversely, the expression of GC1 α and β subunits in COS-7 cells and the endogenous expression of Trx1 were assayed by Western blot (Figure S2c).

### 2.6. Peptide Treatments and Assays of Reduction in Oxidized GC1 by Reduced Trx1 and of Oxidized Trx1 by Reduced GC1, in a Purified System

One μg of recombinant human GC1 was treated with 10 mM DTT at 37 °C for 30 min in the dark or with 100 μM diamide on ice for 30 min in the dark to generate reduced (rGC1) and oxidized GC1 (oGC1), respectively. Four hundred ng of recombinant human Trx1 was treated with 10 mM DTT at 37 °C for 30 min in the dark or with 100 μM diamide on ice for 30 min in the dark to generate reduced (rTrx1) and oxidized Trx1 (oTrx1), respectively. The proteins were transferred onto Amicon Ultra-0.5 centrifugal filters, centrifuged at 14,000× *g* for 10 min, and washed 3 times with a buffer containing 50 mM HEPES, pH 8.0, and 5 mM EDTA to remove excess reagent. The proteins were recovered by flipping the filters and centrifuging at 1000× *g* for 2 min. Various combinations of reduced or oxidized forms of GC1 and Trx1 with or without peptides were mixed and incubated at room temperature for 30 min in the dark. In each tube, a GC1:Trx1 molar ratio was estimated to be 1:5; the concentration of peptides, if added, was 125 μM final. The samples were analyzed by Western blots: gel (12% SDS-PAGE) electrophoresis was conducted under reducing and non-reducing conditions, transferred on nitrocellulose membrane, and then probed for either Trx1 or GC1.

### 2.7. Peptides Treatment of GC1-Trx1 Transnitrosation Reaction in a Purified System

Recombinant human GC1 was treated with 100 μM S-nitrosoglutathione (GSNO) at 37 °C for 30 min in the dark to generate SNO-GC1, as previously described [10]. Recombinant human Trx1 was treated with 10 mM DTT at 37 °C for 30 min in the dark or with 100 μM diamide on ice for 30 min in the dark to generate rTrx1 and oTrx1, respectively, and excess reagents were removed as above. oTrx1 was treated with 100 μM GSNO at 37 °C for 30 min in the dark to generate SNO-oTrx1, as previously described [10]. The proteins were transferred onto Amicon Ultra-0.5 centrifugal filters, centrifuged at 14,000× *g* for 10 min, and washed 3 times with a buffer containing 50 mM HEPES, pH 8.0, and 5 mM EDTA to remove excess reagent. The proteins were recovered by flipping the filters and centrifuging at 1000× *g* for 2 min. Various combinations of SNO-GC1, oTrx1, or rTrx1 with or without peptides were mixed (GC1:Trx1 molar ratio was estimated at 1:5, and the final concentration of each peptide was 125 μM) and incubated at room temperature for 30 min in the dark. The samples were analyzed by biotin or biotin/avidin switch assays as previously described [10], followed by Western blotting. To detect biotinylated proteins, the samples were electrophoresed under non-reducing conditions and probed with the anti-biotin antibody (1:3000, Abcam, Cambridge, UK); the inputs (starting material) were electrophoresed under reducing conditions and probed for Trx1 and GC1.

### 2.8. Biotin Switch Assay and Avidin Enrichment

Free thiols of proteins were blocked in a lysis-blocking buffer containing 50 mM Tris, pH 7.5, 150 mM NaCl, 1% Triton X-100, 1 mM EDTA, 2% SDS, 0.1 mM neocuproine, 0.2 mM PSMF, and 40 mM *N*-ethylmaleimide (NEM) at 50 °C for 30 min in the dark. Excess NEM was removed using cold acetone precipitation. The protein pellets were generated by centrifuging at 14,000× *g* for 10 min at 4 °C and washing 3 times using cold acetone. The pellets were resuspended in a 300 μL buffer containing 25 mM HEPES, pH 7.7, 1 mM EDTA, and 1% SDS, supplemented with 0.1 mM biotin-HPDP, 10 mM ascorbate, and 1 μM CuCl, at room temperature for 1 h in the dark. The negative controls were the blocked samples without ascorbate. Excess biotin-HPDP was removed, and biotinylated proteins were precipitated using cold acetone at −20 °C for 1 h, followed by centrifugation at 5000× *g* for 20 min at 4 °C. The pellets were then dissolved in a HENS buffer. For the detection of biotinylated proteins, resuspended samples were mixed with a 4× Laemmli buffer without β-Mercaptoethanol.

For avidin enrichment, the biotinylated samples were diluted in 700 μL PBS and mixed with 100 μL streptavidin-agarose beads. The mixture was incubated for 1 h at room temperature with regular agitation. The beads were pelleted by centrifuging at 5000× *g* for 10 min and were washed 3 times with 1 mL PBS. The washed beads were mixed with 100 μL of a 1× Laemmli loading buffer with 10% β-Mercaptoethanol and heated at 85 °C for 5 min. The proteins released from the beads were then subjected to Western blotting.

### 2.9. Jurkat T Cells Treatment with Peptides and Etoposide-Induced Apoptosis

Cells in suspension in a 2 mL serum-free medium were treated with either 70% DMSO (20 μL, 0.7% final), scramble peptide, or the inhibitory peptide at a final concentration of 10 μM and incubated at 37 °C for 1 h. After incubation, the cells were washed twice and incubated in a complete medium at 37 °C for 8 h. The cells were then treated with (or without) etoposide (ETO) at 8 μM final at 37 °C for 16 h.

For biotin-avidin switch assays: after 16 h of incubation, the cells were washed twice with cold PBS and lysed in a lysis-blocking buffer containing 50 mM Tris, pH 7.5, 150 mM NaCl, 1% Triton X-100, 1 mM EDTA, 2% SDS, 0.1 mM neocuproine, and 40 mM NEM supplemented with PMSF and a protease inhibitor cocktail. The protein concentration was measured using the Bradford method. Fifty μg of each lysate were analyzed by Western blot as the starting material (inputs), and 200 μg of proteins from each sample were subjected to the biotin switch and avidin assays, as described above. Blots under non-reducing conditions were probed with anti-biotin. Under reducing conditions following avidin assays, blots were probed with anti-GC1 α and β (1:1000), anti-Caspase 3 (1:500), and anti-Trx1 (1:500).

For Caspase-3 activity assays: after 16 h incubation, the cells were washed twice with cold PBS and lysed in the supplier’s 1x lysis buffer (at 10μL per 10^6^ cells). After 15 min incubation on ice, the lysates were centrifuged at 16,000× *g* for 15 min at 4 °C, and the supernatant was collected. Caspase-3 activity was measured following the supplier’s instructions (CASP3C-1KT, Sigma, St. Louis, MI, USA) after a 2 and 3 h incubation.

### 2.10. Statistical Analysis

Value outputs are expressed as the average ± SEM with *n* ≥ 3. The comparison studies with GC1 NO-stimulated activity were conducted with a two-tailed Student’s *t*-test, an α level of 0.05 in the purified system, and the use of one-way ANOVA followed by Tuckey’s posthoc test in the cell studies. Caspase-3 activity was measured in three independent experiments (*n* = 3), and each measurement was in duplicate. The means and standard deviation of each measurement were calculated, and the variance was compared using one-way ANOVA followed by Sidak’s multiple comparison tasks with *p* < 0.05 using GraphPad Prism v9.4.1. *p* < 0.05 was considered statistically significant.

## 3. Results

We previously demonstrated that C610 of the α subunit of GC1 (αC610) was key to the interaction between GC1 and Trx1 under reducing and oxidizing conditions [2,10]. To gain insight into the function of the two NO signaling pathways initiated by either the reduced or the oxidized GC1/Trx1 complex, i.e., enhanced NO-cGMP forming activity or transnitrosation activity, we designed three peptides, overlapping the amino sequence containing αC610, to disrupt the GC1/Trx1 interaction. The peptide sequences are provided in the Supplementary Information (Figures S1 and S2).

### 3.1. Peptides Predicted to Disrupt the Interaction between GC1 and Trx1 Blunt the Ability of Trx1 to Enhance NO-Stimulated Activity of GC1

**Figure 1:**
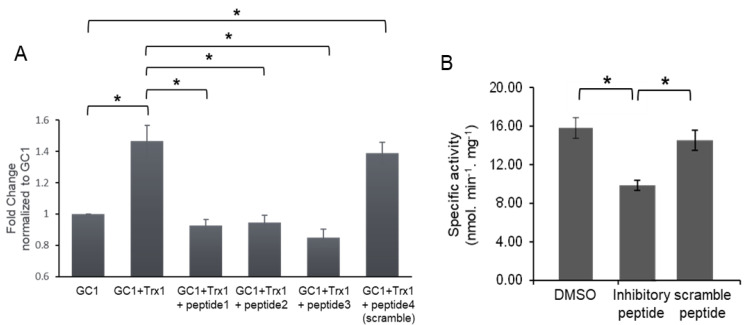
Inhibitory peptides block Trx1-dependent enhancement of NO-stimulated GC1 activity in an in vitro system (**A**) and cells (**B**). (**A**), Purified GC1 and Trx1 (molar ratio 1:7) were mixed in the absence or presence of 25 μM for each peptide, GC1 activity was measured under basal (not shown) and stimulated (1 μM DEA-NO) conditions. Specific activity in nmol cGMP·min^-1^·mg^-1^ (Figure S3) was normalized against GC1 activity and expressed as fold change; *n* = 4 independent experiments, measured in duplicate; * *p* < 0.05. (**B**) COS-7 cells were transfected with GC1 and treated with 10 μM of peptide 3 (inhibitory peptide) and scramble peptide modified by the addition of a cell-penetrating peptide and the fluorophore FITC (Figure S2a). Peptide penetration and GC1 expression were verified by imaging and Western blotting (Figures S2b,c). *n* = 4 independent experiments, measured in duplicate; * *p* < 0.05. The inhibitory peptide had no effect under basal conditions (Figure S2d).

We first screened three peptides and a scramble peptide as a control, and assayed their ability to block the Trx1-enhancing effect on GC1 activity. We used an in vitro system by mixing purified Trx1 and GC1 under reduced conditions. GC1 and Trx1 were mixed at a molar ratio of 1:7, while peptides were added to GC1/Trx1 at a molar ratio of GC1:peptides of 1:800. Similarly, GC1 alone was mixed with the four different peptides in the absence of Trx1 to control for the direct effect of peptides on GC1. Different combinations were kept on ice and in the dark for 20 min, then GC1 activity was assayed under basal and NO stimulated conditions (1 μM DEA-NO). Figure 1A first confirmed the increased NO-stimulated activity of GC1 by Trx1 and showed that the Trx1-dependent increase in NO-stimulated GC1 activity was similarly blunted by the three peptides, while the scramble peptide had no effect. Importantly, none of the peptides affected the NO-stimulated activity in the absence of Trx1 (Figure S4), suggesting that the peptides did not affect the structure of the catalytically active conformation of GC1 but rather affected the functional interaction between GC1 and Trx1. Under basal conditions, Trx1 did not stimulate GC1 activity, as previously observed in cells [2], and conversely, the peptides had no effect (not shown). We next assessed whether these peptides could affect Trx1 enhancement of GC1 activity in a cellular context. We selected one inhibitory peptide (peptide 3) and modified it, together with the scramble peptide, to make the cell-permeable and fluorescent (see the sequence in Figure S2a).

We confirmed the successful addition of the peptide in the cytosol by fluorescent imaging (Figure S2b). We used COS-7 cells, which do not express GC1 but had a detectable level of Trx1, and transfected them with the GC1 expressing vector (Western blot, Figure S2c) and then added 10 μM of inhibitory or scramble peptides or DMSO. Cell lysates were then assayed for GC1 activity. As shown in Figure 1B, the addition of the inhibitory peptide significantly decreased NO-stimulated GC1 activity compared to the control (DMSO) and the scramble peptide. The peptides had no effect on basal activity (Figure S2d). We concluded that this inhibitory peptide in vitro and in cells interfered with the GC1/Trx1 interaction, in turn blunting the enhancing effect of Trx1 on NO-stimulated GC1 activity.

In our previous studies [2,3], we proposed that Trx1 enhances NO-stimulated activity by reducing the thiol oxidation of GC1. We sought to test this hypothesis by determining whether reduced Trx1 (rTrx1) could reduce oxidized GC1 (oGC1) and if this effect was abrogated by the inhibitory peptide.

### 3.2. Bidirectional Reduction of oGC1 by rTrx1 and oTrx1 by rGC1 Is Disrupted by the Interface-Inhibitory Peptide

**Figure 2:**
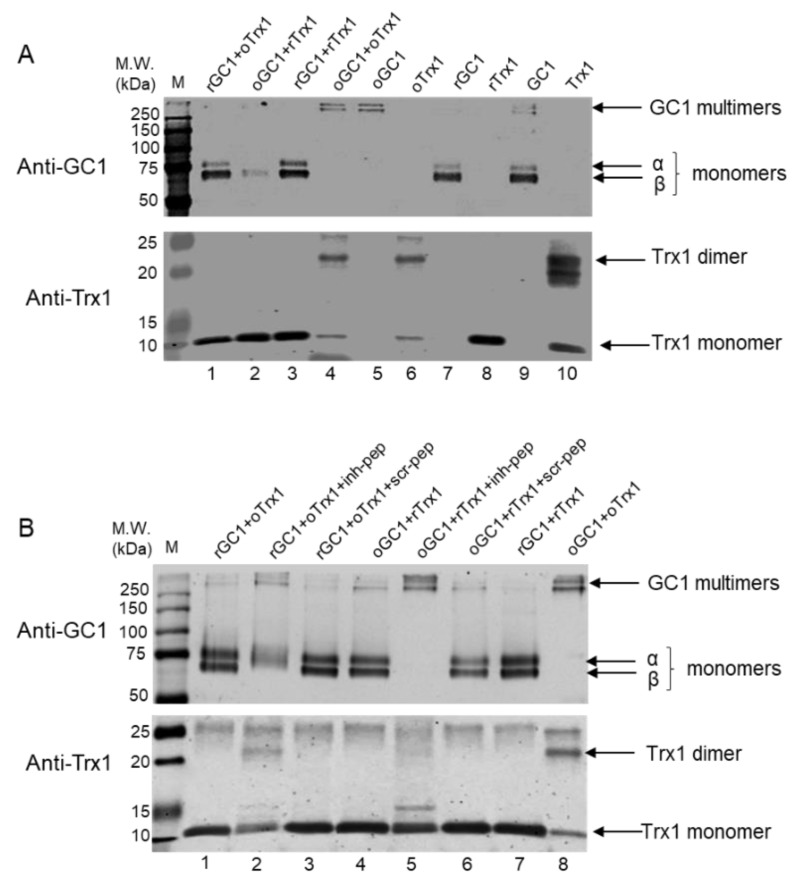
Representative Western blots showing reduced activity of rGC and rTrx1 toward oTrx1 and oGC1, respectively, and blocking of these activities by the inhibitory peptide. (**A**), Purified GC1 and Trx1 were oxidized or reduced as described in Methods and the various oxidized and reduced forms were mixed (molar ratio 1:5) as indicated on the top of the blots (lanes 1–4), then separated by electrophoresis on a non-reducing 12% SDS-PAGE gel. After transfer, the blots were probed with GC1 anti-α and anti-β (upper panel), and anti-Trx1 (lower panel) antibodies. The corresponding reducing gel is provided in Figure S5. Lane 5–8 are the oxidized and reduced forms of Trx1 and GC1, lanes 9 and 10 are untreated GC1 and Trx1. M.W. for Molecular Weight markers. *n* = 3 independent experiments. (**B**)**,** The oxidized and reduced forms of Trx1 and GC1 were mixed as above in the presence of DMSO (lane 1 and 4), 125 μM inhibitory peptide (inh-pep, lane 2 and 5) or scramble peptide (scr-pep, lane 3 and 6), before being transferred and probed as in 2A. Lane 7–8 are controls of reduced or oxidized forms of GC1 and Trx1 mixed together. The corresponding reducing gel is provided in Figure S6. *n* = 3 independent experiments.

Using non-reducing electrophoresis, we first assayed the ability of rTrx1 to reduce oGC1. As a control, we also assayed the effect of reduced GC1 (rGC1) on oTrx1. GC1 and Trx1 were both oxidized using 100 μM diamide and reduced using 10 mM DTT. After removing the excess of reductants and oxidants, the different combinations were mixed (molar ratio GC1:Trx1 was 1:5) and incubated in the dark at room temperature for 30 min before being subjected to non-reducing and reducing electrophoreses. Figure 2A of the non-reducing gel probed for GC1 and Trx1 indicated that the oxidation of GC1 induced the conversion of α and β monomers of GC1 into high molecular weight multimers (compared lane 9 with lane 5, upper panel Figure 2A), while the oxidation of Trx1 led to the conversion of the majority of its reduced monomers into dimers (compared lane 8 to lane 6, lower panel Figure 2A), as previously observed [13,14]. Lanes 9 and 10 were untreated GC1 and Trx1, respectively, both showing mixed monomer/multimer forms.

As anticipated, rTrx1 converted oGC1 multimers into monomers to some extent (lane 5 vs. lane 2, upper panel; though it is better seen in Figure 2B, upper panel lane 4), and unexpectedly, rGC1 converted most of the oTrx1 dimer into Trx1 monomers (lane 6 vs. lane 1 lower panel of Figure 2A). The reduced gel of the same reactions is provided in Figure S5. The ability of each rGC1 and rTrx1 to reduce their oxidized counterparts of the complex confirmed that these reactions were driven by the redox state of both molecules. In addition, our observation that rGC1 reduced oTrx1, hence potentially restoring Trx1 reductase activity, is novel and suggests that GC1 is a regulator of the thiol-redox state of cells via the reduction of inactive oTrx1.

Using the same conditions, we next assayed whether the inhibitory peptide could suppress the reductase activity of GC1 toward oTrx1 and vice versa. The oxidation and reduction of GC1 and Trx1 were conducted as above, and the different mixes incubated with 125 μM of the inhibitory peptide (Inh-pep, the original peptide 3 of Figure 1A) or scramble peptide (Scr-pep) for 30 min at room temperature in the dark, or DMSO as the control. Figure 2B confirmed that rGC1 had the ability to convert the oTrx1 dimers into monomers (lane 8 vs. lane 1, anti-Trx1 lower panel), but this effect was mostly blunted by the presence of the inhibitory peptide (lane 2 vs. lane 1, lower panel). As a control, the rGC1-dependent conversation of oTrx1 from dimers to monomers was maintained in the presence of the scramble peptide (lane 3 compared to lane 8, lower panel). Conversely, rTrx1 was efficient in converting the oGC1 multimers into monomers (lane 4 vs. lane 8, upper panel anti-GC1), and this effect was blocked by the inhibitory peptide (compared lane 4 to lane 5, upper panel), and the reducing effect of Trx1 was maintained with the scramble peptide as a control (lane 6, upper panel). The ability of the inhibitory peptide to block the reducing reactions between Trx1 and GC1 not only confirmed the reducing potential of rTrx1 and rGC1 toward oGC1 and oTrx1, respectively but also indicated that this inhibitory peptide is a powerful tool with which to study the physiological relevance of the complex.

### 3.3. Unidirectional Transnitrosation from SNO-GC1 to oTrx1 Is Blunted by the Interface-Inhibitory Peptide

**Figure 3:**
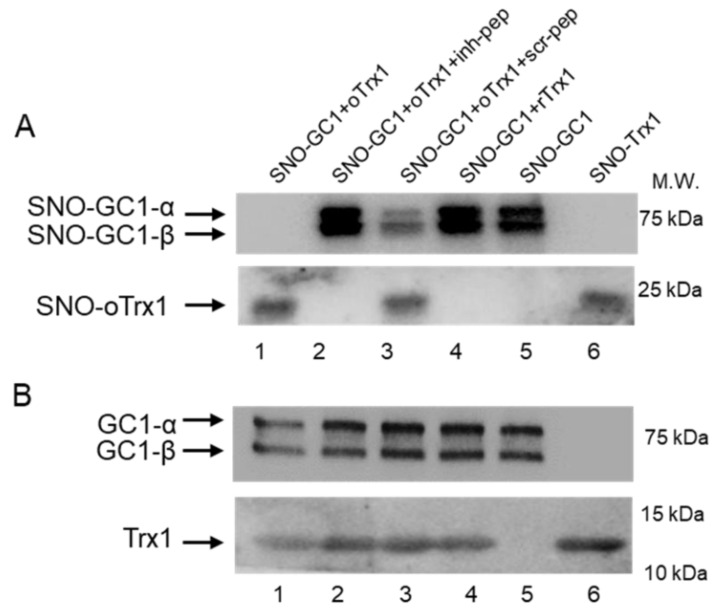
Representative Western blots showing blunted transfer of SNO from GC1 to oTrx1 by the inhibitory peptide. (**A**), Purified GC1 was S-nitrosated with GSNO (SNO-GC1, lane 5); purified Trx1 was oxidized and mixed with SNO-GC1 in the presence of DMSO (lane 1), the inhibitory peptide (lane 2) or the scramble peptide (lane 3). Transfer of SNO from GC1 to oTrx1 (lane 1, DMSO) was blunted by the inhibitory peptide (lane 2) but maintained with the scramble peptide (lane 3). Lane 4 shows that SNO-GC1 did not transnitrosate rTrx1 and lane 5 and 6 were S-nitrosated GC1 and oTrx1 alone. The samples were subjected to biotin switch assays then separated by electrophoresis on a non-reducing 12% SDS-PAGE gel. After transfer, the blots were probed with anti-biotin antibodies (1:3000), to reveal S-nitrosated GC1 (upper panel) and S-nitrosated Trx1 (lower panel). The uncropped Western blots with the “no ascorbate” controls are provided in Figure S7. (**B**), Western blots of the starting materials used in 3A, ran on a reduced 12% SDS-PAGE gel, then transferred and probed with anti-α, anti-β (upper panel), and anti-Trx1 (lower panel) antibodies. Note that in A and B, Trx1 migrates as a dimer and a monomer, respectively, and as expected with a non-reducing (**A**) vs. a reducing (**B**) gel electrophoresis.

The other important function of this complex is to initiate transnitrosation cascades with SNO-GC1, using oTrx1 as a nitrosothiol relay, under oxidative conditions [10]. We first assessed whether the inhibitory peptide could block this transnitrosation reaction, as it involves the transfer of the nitrosothiols group from αC610 of GC1 to C73 of Trx1. As we previously showed, that only the oxidized form of Trx1 (oTrx1) is S-nitrosated, we first oxidized Trx1, then treated oTrx1 or GC1 with GSNO to generate SNO-oTrx1 and SNO-GC1; the latter was mixed with oTrx1. The inhibitory peptide or scramble peptide was added at 125 μM to the SNO-GC1 + oTrx1 mix (the molar ratio GC1:Trx1 was 1:5). The control was the solvent DMSO at 0.9 %. The transfer of the SNO groups between the molecules was assayed by the biotin switch assay under non-reducing conditions. Figure 3A confirmed that SNO-GC1 transnitrosated oTrx1 (lane 1, lower panel) while SNO-GC1 became undetectable (compared lane 1 to lane 5, upper panel), indicating a near complete transfer of the SNO from SNO-GC1 to oTrx1. In contrast, this same transnitrosation reaction was completely blocked by the inhibitory peptide as no SNO-oTrx1 (lower panel, lane 2) and no decrease in SNO-GC1 (upper panel, lane 2) was observed. By contrast, the scramble peptide could not inhibit the transnitrosation reaction between SNO-GC1 and oTrx1, as SNO-oTrx1 was still detected (lane 3 lower panel) while the intensity of SNO-GC1 was drastically reduced (lane 3, upper panel). Of note, SNO-GC1 transnitrosation did not occur with rTrx1 (lane 4), as previously observed [10], confirming that the thiol-redox state is essential in these reactions. Figure 3B is a Western blot of the same samples electrophoresed under reducing conditions.

### 3.4. The Inhibitory Peptide Increases Significantly Caspase-3 Activity in Response to Etoposide While Decreasing S-Nitrosation of Both Caspase-3 and Trx1 in Jurkat T Cells

**Figure 4:**
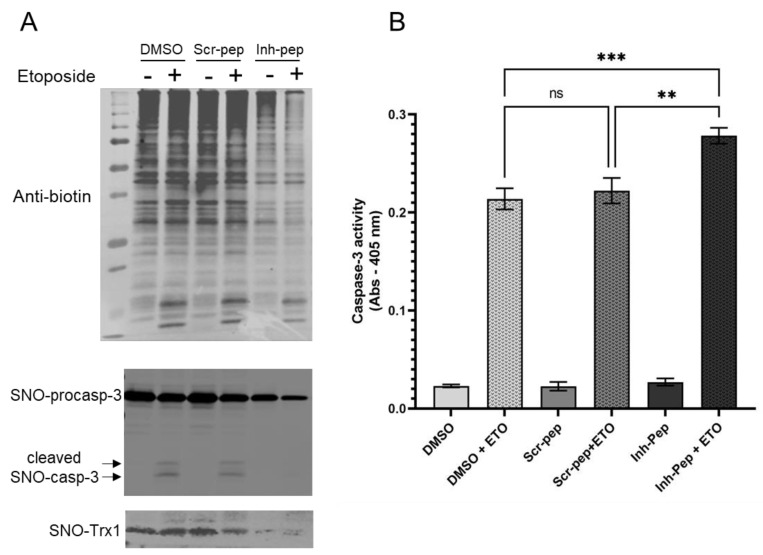
Inhibitory peptide by reducing S-nitrosation in Jurkat T cells increases etoposide-dependent caspase-3 activity. (**A**), Representative Western blots of biotin switch assays of Jurkat T cells ± etoposide (ETO) treated with DMSO, scramble peptide (Scr-pep), or inhibitory peptide (Inh-pep). Upper panel is a blot of a non-reducing gel probed with anti-biotin showing the levels of S-nitrosation in Jurkat cells under different conditions (left lane contains the M.W. markers). Lower panels indicate the levels of S-nitrosated pro-caspase-3, cleaved caspase-3 and Trx1 after avidin purification of the biotinylated samples from the upper panel. *n* = 3; (**B**) Caspase-3 activity was measured with the same sample’s treatment of Figure 4A. The experiments, measured in duplicate, were repeated independently 3 times. ** *p* < 0.001; *** *p* < 0.0005. For clarity, the highly significant (*p* < 0.0001) increase in apoptosis with ETO under the three conditions is not shown. ns: not significant.

The above experiments were conducted in a purified system; thus, we sought to determine whether the disruption of the GC1-Trx1 transnitrosation complex could have biological relevance in a specific cell type. We used the proliferative Jurkat T cells (human leukemic T cell line) because it was shown that S-nitrosation of procaspase-3 by oTrx1 partially blocked the cleavage into active caspase-3 and hence, apoptosis [15,16]. The source of SNO-Trx1 in these cells was unknown and mutational analysis identified C73 as the SNO donor of oTrx1 (to the acceptor C163 of procaspase-3). Since GC1 was expressed in the Jurkat T cells (Figure S8 and [17]), we tested the idea that SNO-GC1 is a source of SNO-Trx1. If so, the GC1/Trx1 complex inhibitory peptide should disrupt the transfer of S-nitrosothiols from GC1 to Trx1 and, hence, the Trx1-dependent S-nitrosation of caspase-3. In turn, an increase in caspase-3 activity and apoptosis in cells treated with the inhibitory peptide is expected. Jurkat T cells were untreated (control, DMSO) or were treated with the scramble peptide (10 μM, 1 h) or with the inhibitory peptide (10 μM, 1 h) and were assayed under basal and apoptotic conditions (etoposide, 8 μM for 16 h). We first conducted biotin-avidin assays to determine the levels of S-nitrosation under various conditions. As shown in Figure 4A, the levels of S-nitrosation were high at the baseline in cells treated with DMSO and the scramble peptide, but there was a visible decrease in global SNO in the cells treated with the inhibitory peptide. Etoposide (ETO) did not affect the overall level of S-nitrosation. The biotinylated samples were then avidin purified, and after electrophoresis, the blots were probed with caspase-3 and Trx1 antibodies (lower panels, Figure 4A). SNO-procaspase-3 was greatly reduced in cells treated with the inhibitory peptide, compared to DMSO and scramble peptide treated cells; S-nitrosation of caspase-3 cleaved fragments was detectable with ETO treatment in the controls (DMSO, scramble peptide) but no SNO-caspase-3 cleaved fragments could be detected in cells with an inhibitory peptide. Conversely, inhibitory peptides strongly decreased the levels of SNO-Trx1 compared to the controls (DMSO and scramble peptide), confirming, in cells, the ability of the inhibitory peptide to block the SNO-GC1 to Trx1 transnitrosation reaction. The corresponding stained Ponceau red and uncropped blots that include controls without ascorbate are provided in Figure S9. Of note, the Western blot of the inputs (cell lysates of the starting material) in Figure S8 shows some reduced procaspase-3 levels of the cells treated with the inhibitory peptides in response to the ETO treatment compared to the controls treated with ETO. Together, this suggests that a lower level of S-nitrosated procaspase-3 resulted in its increased processing in cells where the GC1/Trx1 complex was disrupted. Whether these observations correlated with an increased caspase-3 activity was next assessed with a colorimetric assay based on the cleavage of the substrate DVED under the same above conditions. As shown in Figure 4B, treatment with ETO induced a significant increase in caspase-3 activity compared to samples not treated with ETO, as expected. More importantly, in the Jurkat cells treated with the inhibitory peptide, the apoptotic activity was strongly and significantly increased compared to the cells treated with DMSO or a scramble peptide. We concluded that disrupting the SNO-GC1→oTrx1→procaspase-3 transnitrosation cascade using a peptide inhibitory of the GC1/Trx1 interaction could enhance Jurkat T-cells apoptosis.

## 4. Discussion

The NO-cGMP pathway is a crucial component of vasorelaxation, regulation of blood pressure, cardiac protection, and platelets aggregation, and we showed that the interaction between GC1 and Trx1 supported NO-stimulated GC1 activity and, thus, could be essential for cardiovascular homeostasis. On the other hand, we recently identified the formation of a thiol-oxidized SNO-GC1/oTrx1 complex, which carries transnitrosation cascades. Some of the targets of this oxidized complex are known to be regulated by the NO-cGMP pathway. As oxidative conditions led to the disruption of the canonical NO-GC1-cGMP pathway via heme oxidation and extensive S-nitrosation of GC1, we proposed that the transnitrosation activity of SNO-GC1, amplified by oTrx1, provides an adaptive response to oxidative stress [10].

The chemistry of the interaction between Trx1 and GC1 under oxidized and reduced conditions is different. Under reduced conditions, our previous mutational analyses showed that the association between GC1 and Trx1 involved a mixed disulfide, potentially between the C^32^ of Trx1’s reduced active site and C^610^ of the α subunit of the GC1 heterodimer [2]. By contrast, under diamide-oxidized conditions, oTrx1 was detected as a dimer, as previously observed [13,14]. Previous mass spectrometry (MS) analyses showed that oTrx1’ Cys active site formed a disulfide and C^73^ was amenable to S-nitrosation [18], indicating that oTrx1 lost its reductase activity but gained a transnitrosation activity. We successfully characterized the interaction between oTrx1 and SNO-GC1, revealing a unilateral transfer of S-nitrosothiols from SNO-GC1 to oTrx1 and identifying the C^610^ of the α subunit as a major “SNO donor” (to the Trx1-C^73^ SNO acceptor). In this oxidized complex, the interaction with Trx1 could not involve a mixed disulfide with the C^32^ of Trx1, as C^32^ was engaged in a disulfide with C^35^. To gain insight into these various complexes and their relevance, we used an inhibitory peptide for the interaction. This allowed us to avoid the potential activity/conformational alterations generated by mutations of GC1 or Trx1. The downside of not using Trx1 mutants as previously [2] meant that our ability to “trap” this complex was lost, as was our analysis via the pull-down of this transient complex. The peptides were designed around C^610^ of the α subunit to theoretically block both the mixed disulfide of the GC1/Trx1 reduced complex and the S-nitrosothiol transfer between SNO-C^610^ and C^73^ in the oxidized complex.

We showed in vitro that these C^610^-mimetic peptides blunt the Trx1-dependent enhancement of NO-stimulated GC1 activity without directly affecting NO-stimulated GC1 activity, indicating that the peptides did not alter the conformational/structural properties of the NO-responsive GC1 heterodimer. The efficiency of the peptides and their ability to block the formation of the reduced GC1/Trx1 complex was confirmed in cells after rendering one selected peptide cell permeable. We inferred that the selected peptide (RKINVSPTTYR), which did not contain a disulfide bond unlike the targets of Trx1 reductase activity should not be able to compete with these targets or interfere with the thiol redox regulatory function of Trx1 and the cellular redox state. On the other hand, C^610^ is in a solvent-exposed region of the α subunit of the GC1 heterodimer and has the potential to interact with other proteins. One of these proteins could be a protein disulfide isomerase (PDI), as we previously showed that GC1 interacts with PDI via a mixed disulfide [19]. The consequences of potentially blocking this interaction or others with the inhibitory peptide need to be addressed in future studies.

The assays of the reductase activity of Trx1 and GC1 in the absence and presence of the inhibitory peptide revealed that GC1 has the ability, in vitro, to reduce the Trx1-oxidized dimer. As this reaction was blocked by the inhibitory peptide, it strongly suggests that the disulfide exchange involves αC^610^. In the absence of MS analysis, we did not know what Cys was involved in the Trx1 homodimer. Because of the difference in experimental conditions, the Cys engaged in the homodimer was not unequivocally identified and could be C^69^, C^73^, or/and C^32^ of the active site [13,14,20,21]. As C^610^ is involved in a mixed disulfide with C^32^ of Trx1, and the reduction of Trx1-homodimer by GC1 is blocked by the inhibitory peptide, we speculate that C^610^ could reduce disulfide bonds involving C^32^ in both the active site (C^32^–C^35^) and homodimerization.

GC1 in vivo is found as a dimer, which is sensitive to reducing agents [22], and we proposed that disulfides could have a role in GC1 response to NO [3]. While the diamide-induced multimers of GC1 might not reproduce physiological conditions, our results indicate that rTrx1 can recognize these GC1 multimers and convert them to monomers in an efficient way. Because this Trx1-dependent reduction of GC1 multimers is completely blocked by the inhibitory peptide, this suggests that the multimers formation involves C^610^ of the α subunit, including a potential C^610^ disulfide bond between two GC1 heterodimers. By contrast, we previously showed [10] and confirmed herein that rTrx1 cannot reduce the thiol oxidation of S-nitrosated GC1 (SNO-GC1). Together these results suggest that rTrx1 is not able to interact with SNO-GC1 in vitro.

On the other hand, we confirmed that oTrx1 interacts with SNO-GC1 and demonstrated that the transfer of S-nitrosothiols from SNO-GC1 to oTrx1 is blocked by the inhibitory peptide. We previously identified in vitro other Cys of GC1 that drive a nitrosothiol transfer [10]. The bands of biotinylated SNO-GC1 mixed with oTrx1 are undetectable on the blot of Figure 3, as expected if there is a complete transfer of nitrosothiols; however, in the presence of the inhibitory peptide, the intensity of SNO-GC1 was similar to the controls SNO-GC1, and SNO-GC1 + rTrx1, as if no SNO transfer occurred. This could suggest that the inhibitory peptide blocks the formation of the complex SNO-GC1/oTrx1 rather than the specific SNO-αC^610^ to Trx1-C^73^ transnitrosation reaction. MS analysis will be conducted, in the future, to assess the exact mechanism (due to the transient property of the interaction, we have not been able to do pull-downs).

**Figure 5:**
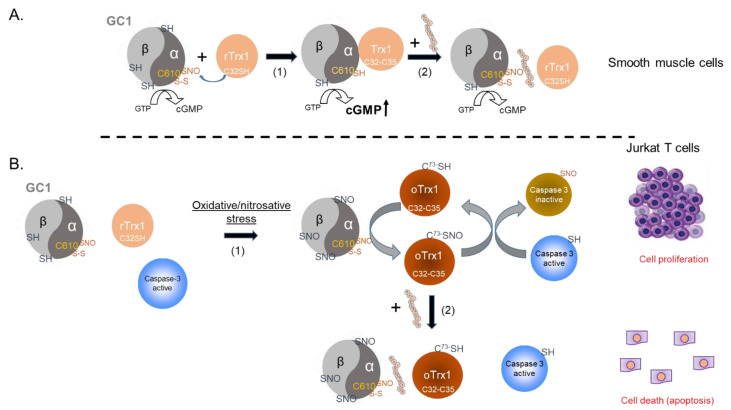
Proposed models of the inhibitory peptide effects on NO-cGMP signaling pathway and SNO transnitrosation cascades. (**A**) Under reducing conditions, in smooth muscle cells for example, rTrx1 and GC1 associate via a mixed disulfide between C610 of the GC1 α subunit and C32 of Trx1 [2] (C610 is endogenously S-nitrosated [25] or/and engaged in a disulfide). In turn, Trx1 increases NO-stimulated GC1 activity thus enhancing the NO-GC1-cGMP pathway (1). Addition of the inhibitory peptide, by blocking the Trx1-GC1 complex formation, blunts Trx1 enhancement of NO-stimulated GC1 activity (2). (**B**) Oxidative and/or nitrosative stress induces multiple S-nitrosation of GC1 and oxidation of Trx1 (oTrx1). In Jurkat T cells, the levels of S-nitrosation are endogenously elevated; a transnitrosation cascade initiated by SNO-GC1 and mediated by oTrx1 leads to caspase-3 S-nitrosation, which renders it inactive leading to cell proliferation (1). In the presence of the inhibitory peptide, SNO-GC1 and oTrx1 interaction is blocked, the transnitrosation cascade is disrupted; thus, caspase 3 remains active, supporting cell death (2).

The addition of the inhibitory peptide to cells under reducing conditions showed that the enhancement of a Trx1-dependent NO-cGMP pathway could be blunted (Figure 5A). This provides us with a tool to directly assess, in the future, the significance of this complex in the vasorelaxation of blood vessels under homeostatic conditions. Conversely, to test the biological, potentially therapeutic relevance of this inhibitory peptide, we thought of blocking GC1/Trx1-dependent transnitrosation under specific oxidative pathophysiological conditions (Figure 5B). We focused on the known link between cancer cell proliferation and the inhibition of caspases activity by S-nitrosation [23,24]. Specifically, we used Jurkat T cells for which Trx1-dependent transnitrosation was shown to inhibit the apoptotic caspase-3 pathway [15,16]. We observed that in the absence or presence of etoposide (ETO), which induces apoptosis, baseline levels of global S-nitrosation were elevated in the controls (DMSO and scramble peptide) and correlated with strong S-nitrosation of procaspase-3 and detectable SNO-Trx1. However, in the presence of the inhibitory peptide, the basal S-nitrosated procaspase-3 levels were drastically reduced, and SNO-Trx1 was barely detectable. When ETO was added, SNO-procaspase 3 levels were slightly reduced with the controls and the inhibitory peptide, suggesting that ETO reduced S-nitrosation of procaspase-3 to some extent but not as pronounced as that of Fas treatment [24]. Conversely, ETO treatment increased significantly caspase-3 activity; importantly, caspase-3 activity was significantly higher in the cells treated with the inhibitory peptide. Together with the decreased S-nitrosation of procaspase-3 and Trx1, compared to the control DMSO and scramble peptide, the increase in caspase-3 activity indicated that the inhibitory peptide efficiently blocked the transfer of nitrosothiol from GC1 to Trx1 and consequently decreased the transnitrosation from Trx1 to procaspase 3. The disruption of the SNO-GC1→Trx1→procaspase3 transnitrosation cascade confirmed that SNO-GC1 was a major source of SNO-Trx1, yet not unique, and importantly, that this mimetic peptide could be a valuable tool to potentially decrease the tumor development associated with excess S-nitrosation (Figure 5B).

In fact, a direct correlation between Trx1 and S-nitrosation has been established in other cancer types [26], including hepatocellular carcinoma [27]. Another promising aspect of this peptide is that under these conditions of nitrosative/oxidative stress, SNO-GC1 cannot interact with rTrx1; thus, the peptide does not interfere with Trx1’s ability to regulate the redox state of the cells, including its denitrosation activity. This could be important considering the dichotomous effect of Trx1 on the development and inhibition of tumor growth in relation to Trx1’s ability to transnitrosate and denitrosate various caspases as a function of its redox state [28]. Nonetheless, we cannot rule out that this peptide, by mimicking part of the α subunit’ Trx1-binding domain, could potentially interfere with other transnitrosation reactions driven by SNO-oTrx1 with its own targets. Interestingly, the GC1 α subunit, which is sufficient to initiate transnitrosation cascades [10], was reported to mediate, independently of the β subunit or cGMP production, prostate and endometrial cancer cell proliferation [29,30]. Together, these reports and our study support the idea that the α subunit of GC1 is a key player in oncogenic processes and in association with oTrx1 and transnitrosation reactions.

## 5. Patent

A provisional patent application was filed.

## Data Availability

All the data are contained within the article and the Supplementary Materials.

## Supplementary Materials

https://www.mdpi.com/article/10.3390/antiox12040906/s1, Figure S1: Sequence of peptides designed to disrupt the GC1/Trx1 complex in a purified system; Figure S2: Peptide designed to disrupt the GC1/Trx1 complex in cells; Figure S3: NO-stimulated specific activity of GC1 ±Trx1 mixed with each peptide; Figure S4: NO-stimulated activity of GC1 is not affected by incubation with the peptides alone; Figure S5: Western blot under reducing conditions of the inputs used in Figure 2A; Figure S6: Western blot under reducing conditions of the inputs used in Figure 2B; Figure S7: Uncropped Western blots from Figure 3A; Figure S8: Input of Jurkat cells used as starting material for biotin/avidin switch assay of Figure 4; Figure S9: Uncropped Western blot and Ponceau red stained blot from Figure 4.
