# Supplementary material for: Inhibitory Peptide of Soluble Guanylyl Cyclase/Trx1 Interface Blunts the Dual Redox Signaling Functions of the Complex
Source: Antioxidants (Basel). 2023 Apr 10;12(4):906. doi: 10.3390/antiox12040906 (PMC10135718; doi:10.3390/antiox12040906)

SUPPLEMENTARY MATERIALS

Inhibitory peptide of soluble guanylyl cyclase/Trx1 interface blunts the dual redox signaling functions of the complex

Chuanlong Cui, Ping Shu, Tanaz Sadeghian, Waqas Younis, Hong Li, Annie Beuve

Figure S1: Sequence of peptides designed to disrupt the GC1/Trx1 complex in a purified system.

Figure S2: Peptide designed to disrupt the GC1/Trx1 complex in cells. a) Sequences of the peptide; b) imaging of peptides penetration in COS-7 cells; c) Western blots of lysates from COS-7 cells transfected with GC1 and treated with DMSO or peptides; d) Basal activity in COS-7 cells overexpressing GC1 is unchanged by the peptides treatment.

Figure S3: NO-stimulated specific activity of GC1 ±Trx1 mixed with each peptide, expressed in $nmol.min^{-1}.mg^{-1}$.

Figure S4: NO-stimulated activity of GC1 is not affected by incubation with the peptides alone.

Figure S5: Western blot under reducing conditions of the inputs used in Figure 2A showing that the starting material was similar in each proteins mix.

Figure S6: Western blot under reducing conditions of the inputs used in Figure 2B showing that the starting material was similar in each proteins combination.

Figure S7: Uncropped Western blots from Figure 3A and including ascorbate controls.

Figure S8: Input of Jurkat cells used as starting material for biotin/avidin switch assay of Figure 4.

Figure S9: Uncropped Western blot and Ponceau red stained blot from Figure 4.

Figure S1: Sequence of peptides designed to disrupt the GC1/Trx1 complex in a purified system. Peptides 1, 2 and 3 were designed in the proximity of C610 (in red). C-terminal region of the GC1 α subunit is shown (human sequence; 100 % identity with rat, mouse and bovine sequences in this region). Peptide 1: (601) VTLANKFESCS (611); Peptide 2: (605) NKFESCSVPRK (615); Peptide 3: (614) RKINVSPTTYR (624); Peptide 4 (scramble): KSRVNPSKCEF. Synthesized by Epoch life.

```
481  TMLFSDIVGFTAICSQCSPLQVITMLNALYTRFDQQCGELDVYKVETIGD  530

531  AYCVAGGLHKESDTHAVQIALMALKMMELSDEVMSPHGEPIKMRIGLHSG  580
                                 1.VTLANKFESCS 3.RKINVSPTTYR
581  SVFAGVVGGKMPRYCLFGNNVTLANKFESCSVPRKINVSPTTYRLLKDCP  630
                                     2.NKFESCSVPRK
631  GFVFTPRSREELPPNFPSEIPGICHFLDAYQQGTNSKPCFQKKDVEDGNA  680

681  NFLGKASGID
```

Figure S2: Peptide designed to disrupt the GC1/Trx1 complex in cells.

Figure S2a. Sequences of the peptide. Previous peptide 3 (yellow highlight) was selected for addition of the fluorophore FITC at the N-terminal followed by a cell penetrating sequence highlighted in blue. The scramble peptide (grey highlight) was similarly modified. Synthesized by Abclonal science.

Inhibitory peptide: FITC-RKKRWFRRRRPKWKKRKINVSPTTYR

Scramble peptide: FITC-RKKRWFRRRRPKWKKKSRVNPSKCEF

Figure S2b: imaging of peptides penetration in COS-7 cells. Imaging of COS-7 cells overexpressing GC1 treated with DMSO (0.7%) or 10 µM inhibitory peptide or scramble peptide. Representative images taken with a Zeiss 200 microscope (20X objective; 800 ms exposure of green fluorescence imaging).

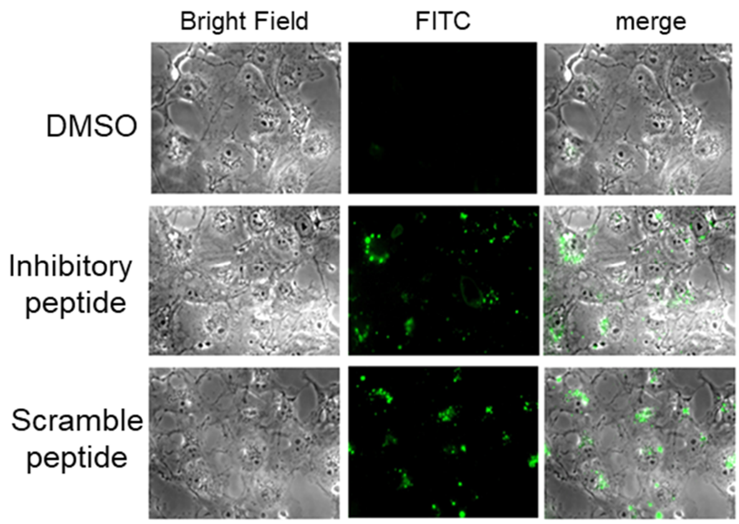

Figure S2c: Western blots of lysates from COS-7 cells transfected with GC1 and treated with DMSO or peptides, showing similar overexpression of GC1 α and β subunits, and endogenous expression of thioredoxin 1. In each lane, 40 µg of lysates were subjected to electrophoresis (12 % SDS-PAGE) and probed with anti-α (1:1000, ThermoFisher), anti-β (1:1000, Cayman) and anti-Trx1 (1:500, Cell signaling) antibodies.

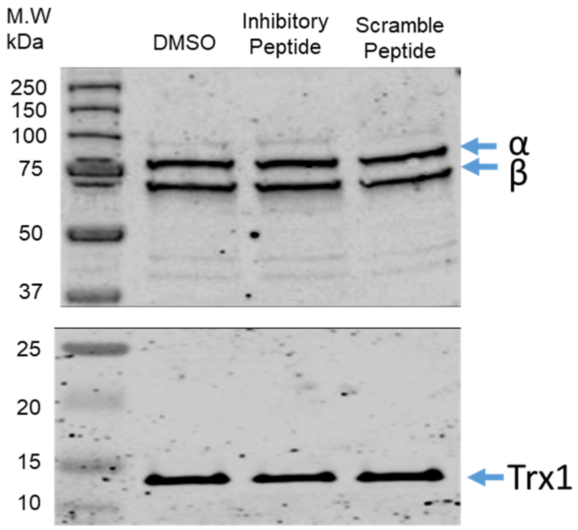

Figure S2d: Basal activity in COS-7 cells overexpressing GC1 is unchanged by the peptides treatment. In contrast to the NO-stimulated GC1 activity, there was no significant difference for the basal specific activity treated with DMSO (control), inhibitory peptide and the scramble peptide (Materials and Methods). *n*=4 independent experiments in duplicate.

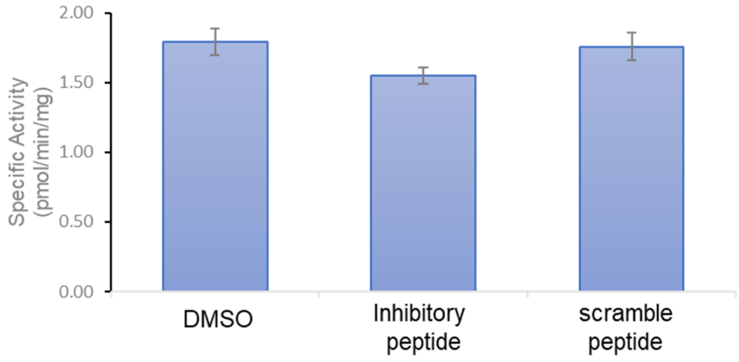

Figure S3: NO-stimulated specific activity of GC1 ±Trx1 mixed with each peptide, expressed in nmol cGMP.min^{-1}.mg^{-1}. These data were used to calculate the fold changes and significance of difference between GC1 and the different mixtures of Fig.1A. DEA-NO was the NO-donor, used at 1 µM final concentration. *n*=4 independent experiments in duplicate, expressed in nmol cGMP.min^{-1}.mg^{-1} ± S.E.M.

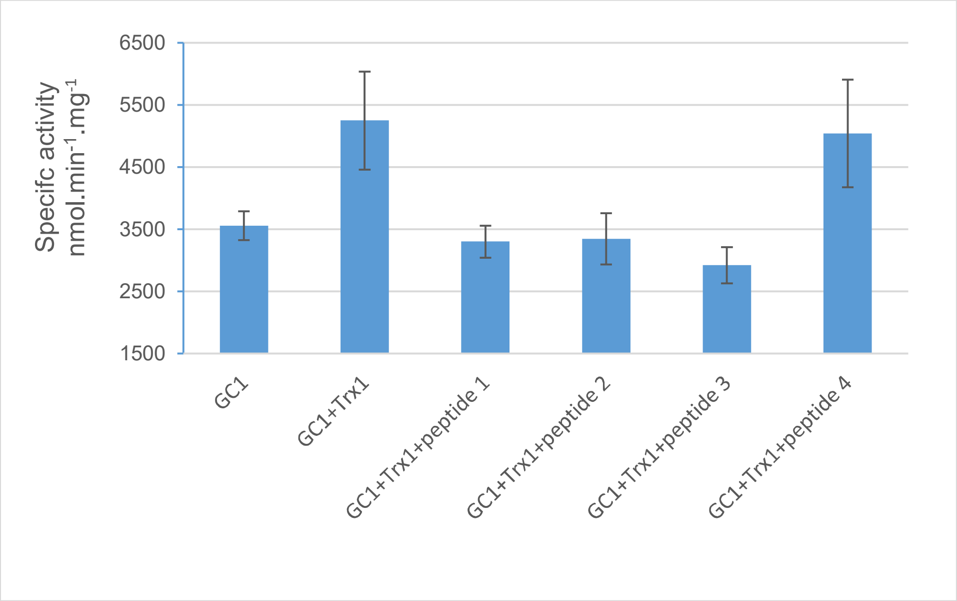

Figure S4: NO-stimulated activity of GC1 is not affected by incubation with the peptides alone. DEA-NO was the NO-donor, used at 1 µM final concentration. n=4 independent experiments done in duplicate. NO-stimulated activity of GC1 was compared to NO-stimulated GC1 activity in the presence of the various peptides in the same in vitro/purified system as above.

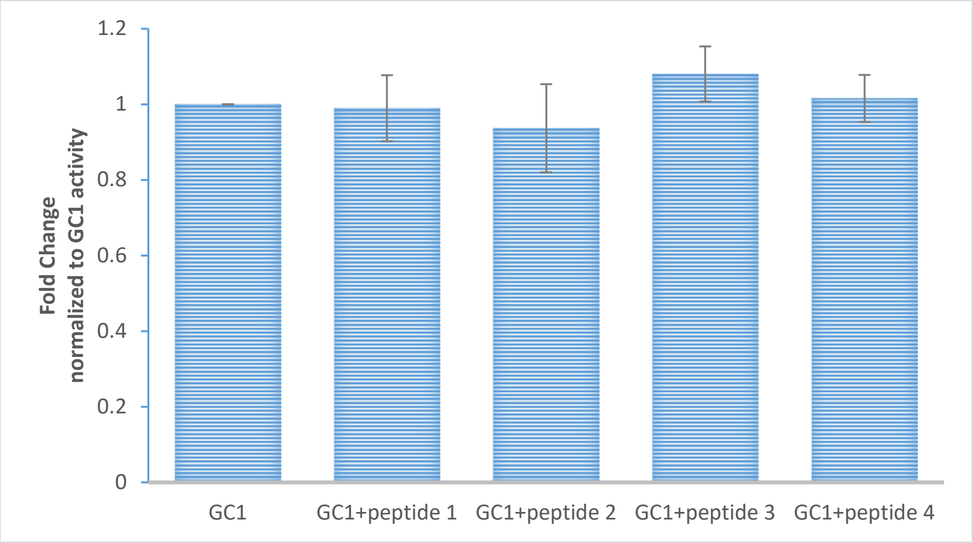

Figure S5: Western blot under reducing conditions of the inputs used in Figure 2A showing that the starting material was similar in each combination._Samples were electrophorated on 12% SDS-PAGE reducing gel, then transferred and probe with anti-α (1:1000, ThermoFisher), anti-β (1:1000, Cayman) and anti-Trx1 (1:500, Cell signaling) antibodies. The system of detection was an Odyssey imager, using fluorescent secondary antibodies.

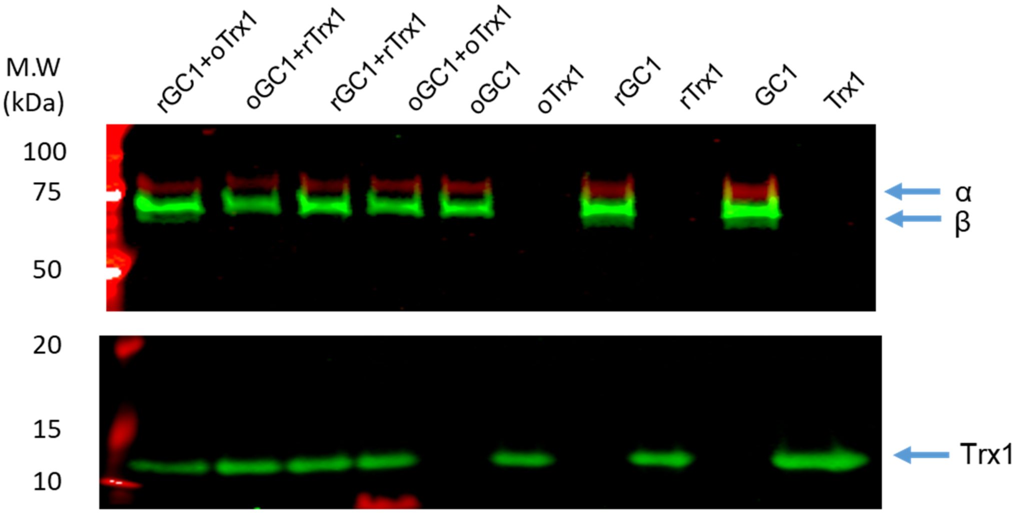

Figure S6: Western blot under reducing conditions of the inputs used in Figure 2B showing that the starting material was similar in each combination. Samples were electrophorated on 12% SDS-PAGE reducing gel, then transferred and probe with anti-α (1:1000, ThermoFisher), anti-β (1:1000, Cayman) and anti-Trx1 (1:500, Cell signaling) antibodies. The system of detection was an Odyssey imager, using fluorescent secondary antibodies.

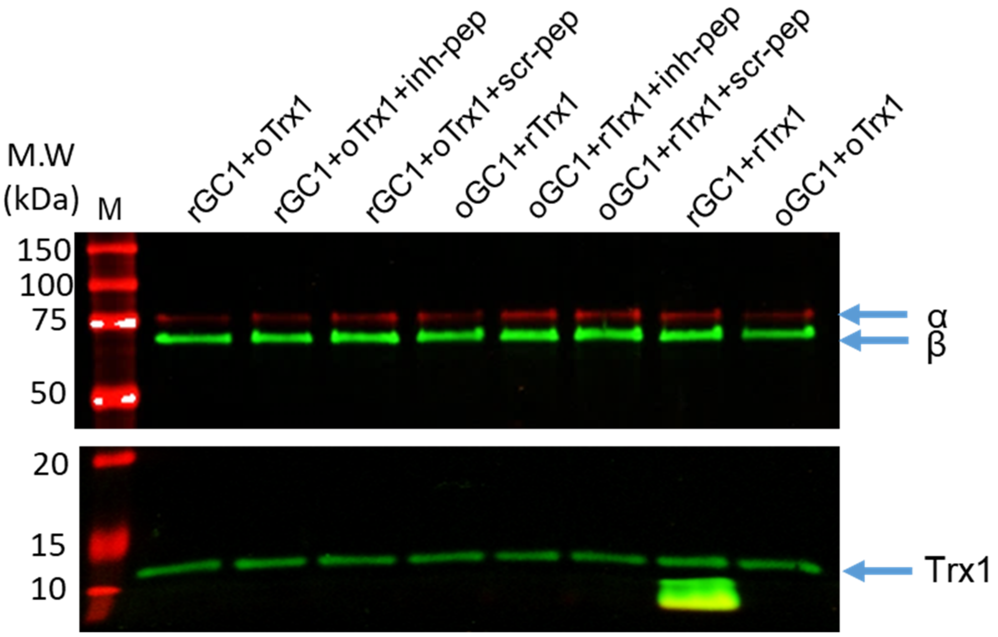

Figure S7: Uncropped Western blots from Figure 3A. Controls of the biotin switch assays were samples not treated with Ascorbate (no Asc). Left is imaging with fluorescent secondary antibodies using an Odyssey imager. Only GC1 was detectable; the blot was thus reprobed with HRP secondary antibodies and Trx1 was detected by enhanced chemiluminescence (ECL).

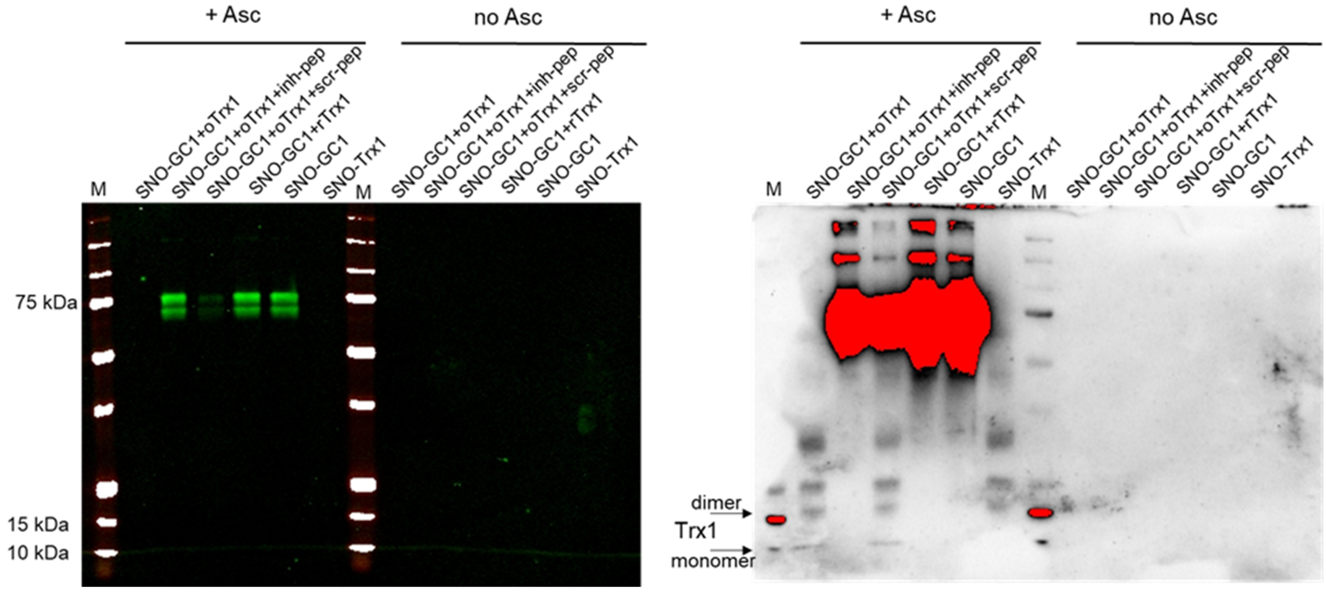

Figure S8: Input of Jurkat cells used as starting material for biotin/avidin switch assay of Figure 4. Left panel: Ponceau red staining of the membrane of electrophorated samples on a 12% reducing gel. Right panel, same blot probed with anti- alpha, anti-beta (Cayman, 1:1000), anti-casp-3 (Novus Biologicals, 1:500) and anti-Trx1 (Cell signaling, 1:1000) antibodies. Note the decreased intensity of procasp-3 signal suggesting increased processing in Jurkat cells treated with etoposide (ETO at 8 µM) and the inhibitory peptide compared to the controls (DMSO, scramble peptide) treated with ETO.

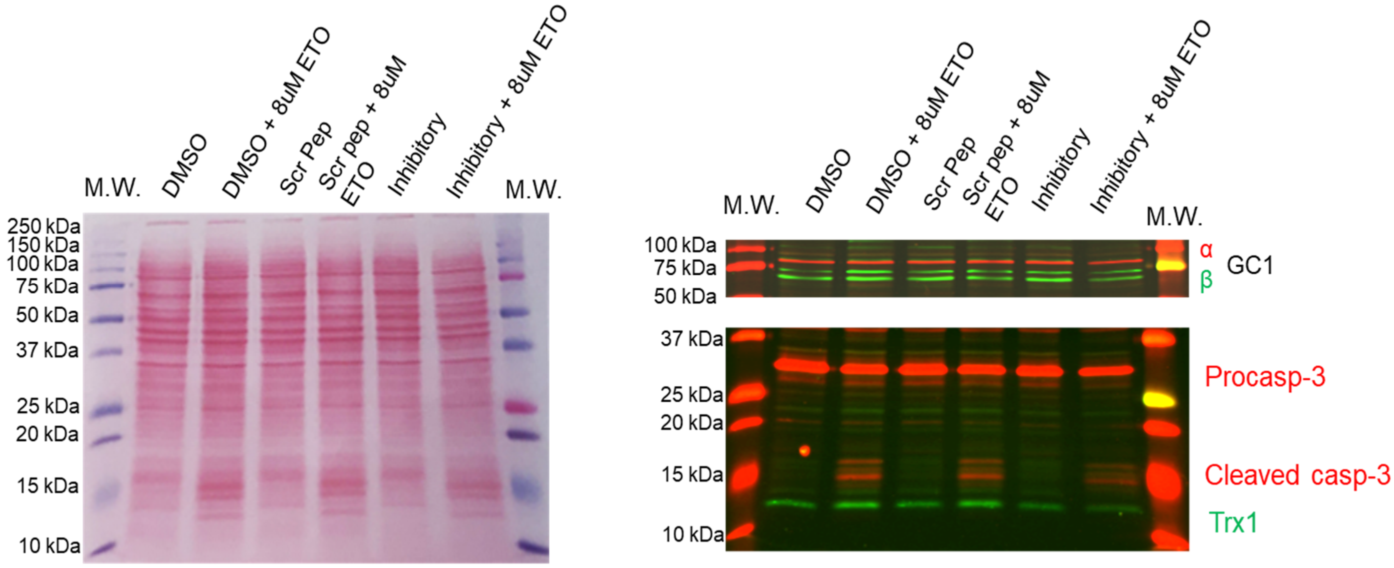

Figure S9: Uncropped Western blots from Figure 4. Samples of the biotin switch assays including samples not treated with Ascorbate (- Asc) were electrophorated on non-reducing gel. Left panel, Ponceau red of the corresponding blots showing similar amount of samples. Right panel, blot was probed with anti-biotin antibodies as described in Methods.

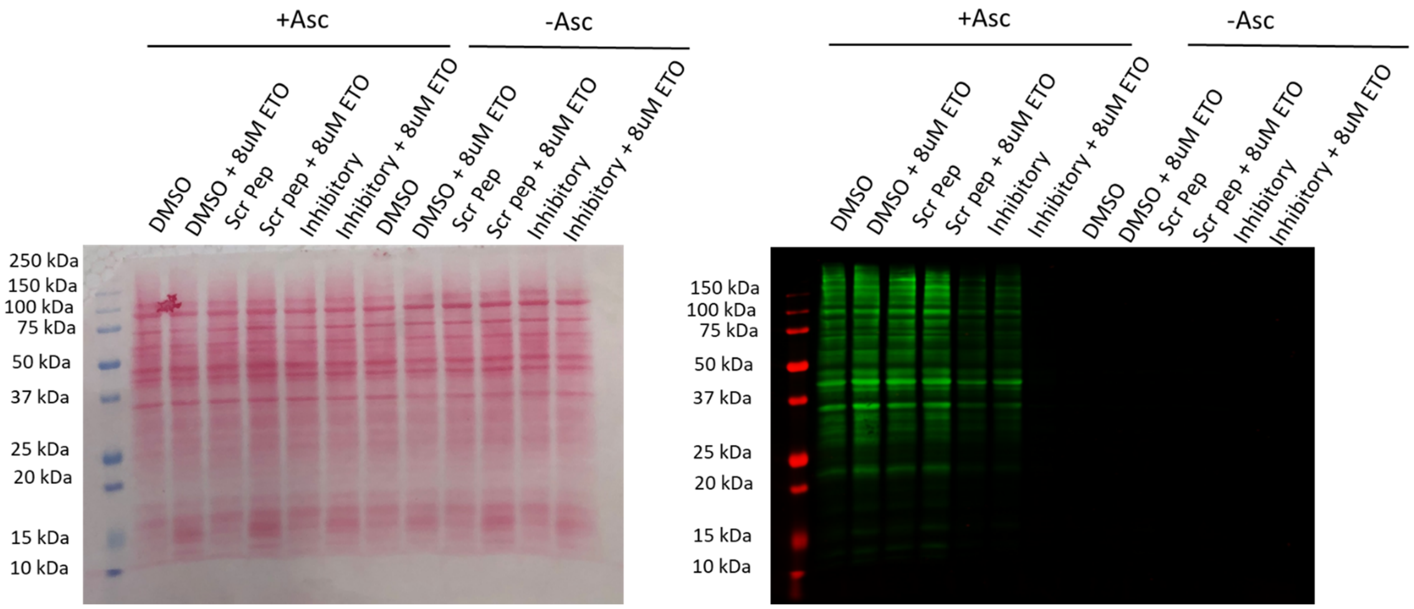

Supplement: Supplementary file 1 [file antioxidants-12-00906-s001.zip › antioxidants-2233114-supplementary.pdf]
